# Supplementary material for: Response Surface Modeling and Optimization of the Extraction of Phenolic Antioxidants from Olive Mill Pomace
Source: Molecules. 2022 Dec 6;27(23):8620. doi: 10.3390/molecules27238620 (PMC9741320; doi:10.3390/molecules27238620)
Supplement: Supplementary file 1 [file molecules-27-08620-s001.zip › molecules-2056978-supplementary.pdf]

Supplementary Material S1

Response surface modeling and optimization of the extraction of phenolic antioxidants from olive mill pomace

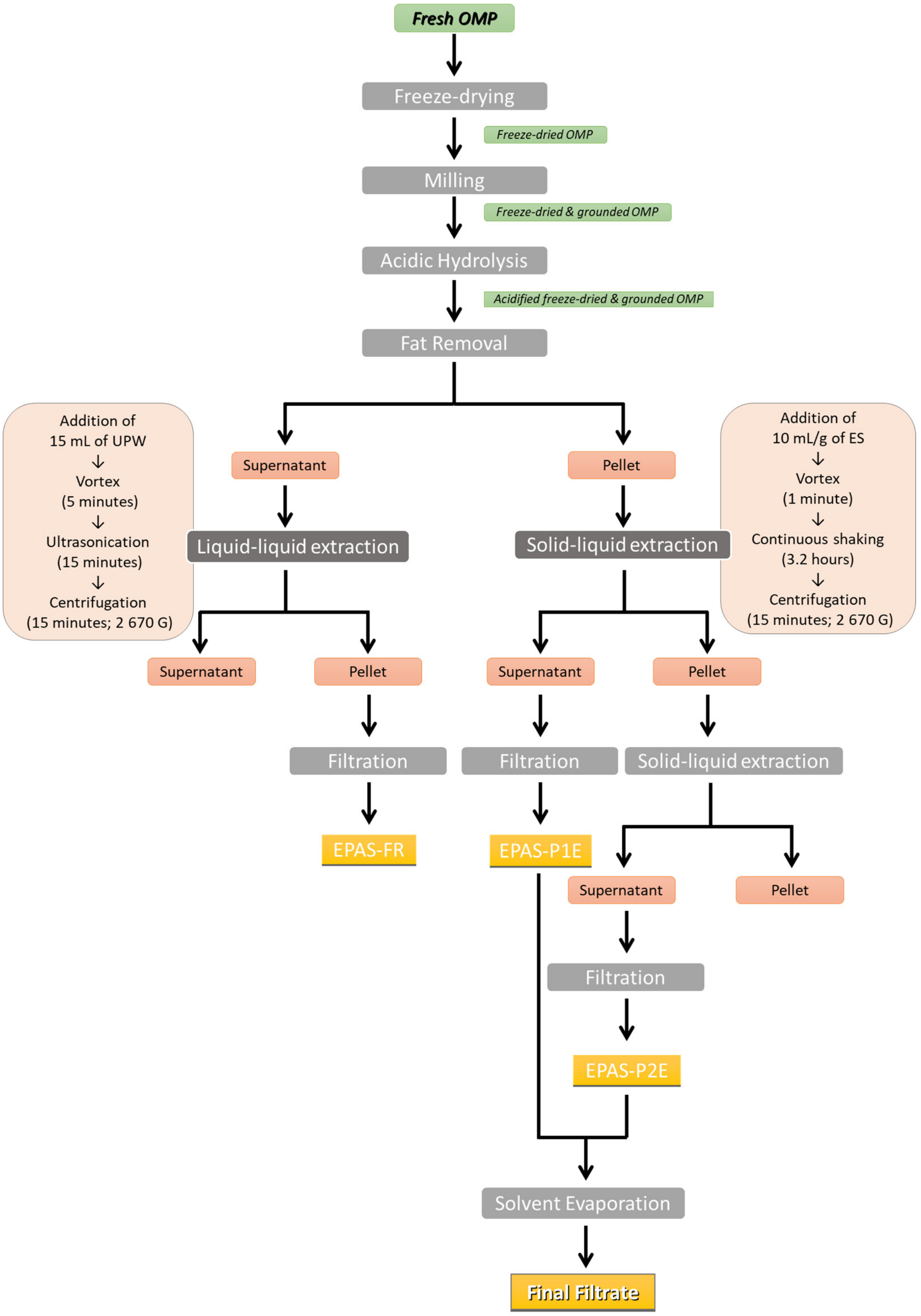

Figure S1. Optimized two-step solvent extraction for the recovery of phenolic antioxidants from olive mill pomace samples (ES – Extraction Solvent; OMP – Olive Mill Pomace; EPAS-FR – Extracted Phenolic Antioxidants during the Fat Removal procedure; EPAS-P1E – Extracted Phenolic Antioxidants during the primary extraction; EPAS-P2E – Extracted Phenolic Antioxidants from the secondary extraction procedure; UPW – Ultrapure water).
